# A piecewise constitutive model for collagen fiber tissues

**DOI:** 10.1007/s10856-022-06660-9

**Published:** 2022-04-28

**Authors:** X. L. Ji, H. H. Zhang, S. Y. Han

**Affiliations:** 1 School of Civil Engineering and Architecture, Nanchang Hangkong University, Nanchang, Jiangxi 330063 PR China; 2 State Key Laboratory for Strength and Vibration of Mechanical Structures, Xi’an, Shaanxi 710049 PR China

## Abstract

Inspired by Meyers et al. (*Science*, 2013), a piecewise model is established so as to individually predict both the heel region and the linear region of stress–strain curve. When the piecewise model satisfactorily predicts the experimental data, the constitutive parameters are precisely identified with definite physical significances. Along with this piecewise guideline, a complete constitutive model can be established for the whole stress–strain curve of collagen fiber tissues with the failure region as well.

**Figure:**
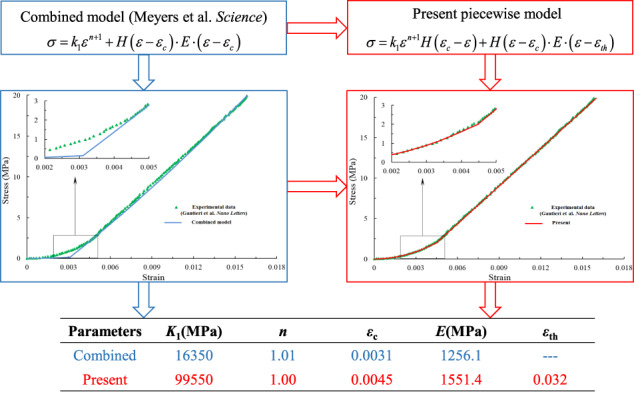
Graphical abstract

Graphical abstract

## Introduction

Soft tissues [1] include tendon, ligament, muscle and nerve. They connect, support, or surround other structures and organs of the body. Actually, fibers are the most frequent motifs in the design of natural tissues [2]. As one type of strong fibers, collagen fiber is composed of proteins with long triple-helical collagen molecules [3, 4]. Essentially through type-I collagen fibers, tendon and ligament are formed as two typical collagen fiber-based soft tissues [5].

**Fig. 1:**
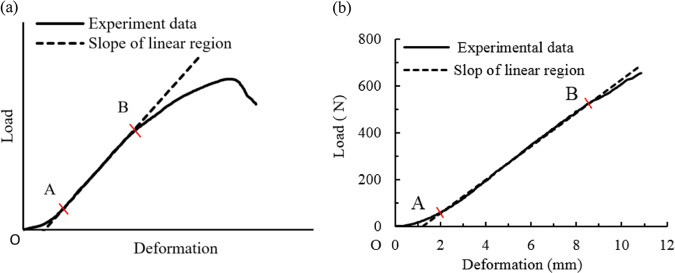
Typical load-deformation curves for collagen fiber-based tissues. **a** For a rabbit limb tendon [8]. **b** For the bone medial collateral ligament of dog [9]

Mechanical tests on collagen fiber-based tissues showed that there is a distinct heel region (i.e., OA) ahead of the principal linear region (i.e., AB) [5–9], as shown in Fig. 1a for the rabbit limb tendon [8] and in Fig. 1b for the bone medial collateral ligament of dog [9]. Gautieri et al. [4] revealed that the heel region is due to straightening of twisted triple-helical molecules at small strains while the linear region is followed by axial stretching and eventual molecular uncoiling. Buehler and Wong [10] thought that entropic elasticity dominates at small deformation and then transmits to energetic elasticity at large deformation, and hence, deformation was characterized by first straightening and breaking of hydrogen bonds in the heel region, followed by stretching of covalent bonds in the protein backbone in the linear region. Based on X-ray scattering experiment of rat tail tendon, Fratzl et al. [11] found that, in the heel region, crimp of the fiber was removed, corresponding to straightening of molecular kinks in the gap, while the linear region was the gliding of collagen fiber molecules. Overall, the heel region is strongly nonlinear with a complex deformation mechanism in soft tissues due to the removal of triple-helical structures, sliding and recruitment while the principal region is linear because of the stretching of collagen fibers backbones.

Thus, to understand the mechanical behavior of soft tissues, it is of great interest to model the two regions.

## A piecewise constitutive model

In [12], by considering the two regimes of unfurling and straightening of polymer chains and stretching of the polymer chain backbones, a combined model was suggested as^1^

1 $$\begin{array}{c}\sigma = k_1\varepsilon ^{n + 1} + H\left( {\varepsilon - \varepsilon _c} \right) \cdot E \cdot \left( {\varepsilon - \varepsilon _c} \right)\\ = {\rm{term}}1 + {\rm{term}}2\end{array}$$

where *H*(*ε* − *ε*_*c*_) is the Heaviside function, and *k*_1_, *n*, *ε*_c_ and *E* are material parameters. As explained in [12], *ε*_c_ is the characteristic transition strain from the heel region to linear region greater than which collagen fibers are fully stretched.

Mathematically, for the linear region with strains greater than *ε*_c_, the slope of Eq. (1) is

2 $$\frac{{d\sigma }}{{d\varepsilon }} = k_1\left( {n + 1} \right)\varepsilon ^n + E$$

Equation (2) indicates that, due to the effect of first term, *E* in the second term is not the modulus of the linear region, unlike explained in [12].

To suppress this effect, the opposite Heaviside function *H*(*ε*_*c*_ − *ε*) is needed as a multiplier for the first term. Thus, the two terms are activated in the heel region for strains less than *ε*_c_ and in the linear region for strains greater than *ε*_c_, respectively.

On the other hand, to ensure the continuity *ε*_c_, an extra quantity *ε*_*th*_ is introduced in the second term, which is actually the intercept strain of the linear region in geometry, and physically interpreted as the threshold strain included in the three-parameter Weibull distribution [13–15].

It turns out that we have the piecewise constitutive relation as

3 $$\sigma = k_1\varepsilon ^{n + 1}H\left( {\varepsilon _c - \varepsilon } \right) + H\left( {\varepsilon - \varepsilon _c} \right)E\left( {\varepsilon - \varepsilon _{th}} \right)$$

It should be mentioned that the five parameters in Eq. (3) have definite physical significances.

## Results and comparisons

For experimental curves of collagen fiber tissues with the heal region and the linear region, several other models have been proposed. In this section, the piecewise constitutive model is utilized to predict experimental data and then compared.

### For the bovine Achilles tendon

**Fig. 2:**
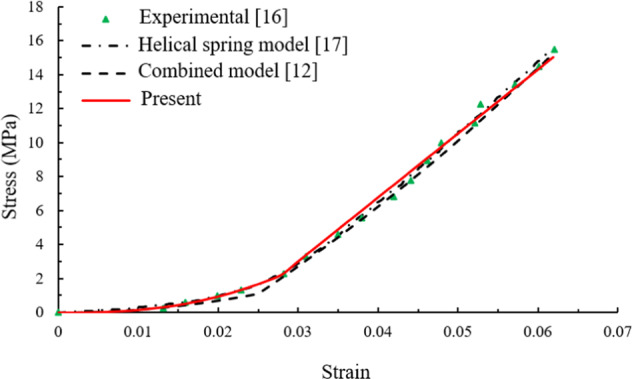
Constitutive behavior of bovine Achilles tendon

Dependence of stress on strain for a bovine Achilles tendon was obtained by means of the X-ray diffraction method [16], as the green dots shown in Fig. 2.

This curve was even studied by using the helical spring model in [17], and can also be predicted by using the combined model in Eq. (1) and the piecewise constitutive relation in Eq. (3). By using the least mean square (LMS) algorithm, the whole accuracy of identified constitutive parameters with experimental results is controlled by correlation coefficient $R^2 = \mathop {\sum}\nolimits_i {\left( {D_i - \bar y} \right)} /\mathop {\sum}\nolimits_i {\left( {y_i - \bar y} \right)^2}$, where *D*_*i*_ and *y*_*i*_ is the *i*th data point of identified and experimental value, and $\bar y = \frac{1}{n}\mathop {\sum}\nolimits_i^n {y_i}$ is mean value of experimental value. The constitutive parameters are identified for different models and tabulated in Table 1.

**Table 1:** The identified parameters for the bovine Achilles tendon

Parameters	*k*_1_ (MPa)	*n*	*ε*_c_	*ε*_th_	*E* (MPa)
Helical Spring model	–	–	0.026	–	426
Combined model	2330	1.02	0.025	–	194.8
Present	22,820	1.58	0.028	0.022	377.2

The modulus is 426 MPa for the helical spring model in [17], 194.8 MPa for the combined model, and 377.2 MPa for the piecewise relation. Compared with the reference value of 400 MPa from the experiment [16], the piecewise relation gives a more accurate modulus than the others.

In the helical spring model, the critical strain *ε*_*c*_ = 437/426–1 = 0.026 through Eq. (41) in [17]. The value is *ε*_*c*_ = 0.028 for the piecewise relation and *ε*_*c*_ = 0.025 for the combined model. These three values are almost same.

However, from Table 1, the differences are over 50% for parameter *n* and nearly ten times for parameter *k*_1_ between the piecewise relation and the combined model.

Overall, the piecewise model is better than the others.

### For the rabbit medial collateral ligament

**Fig. 3:**
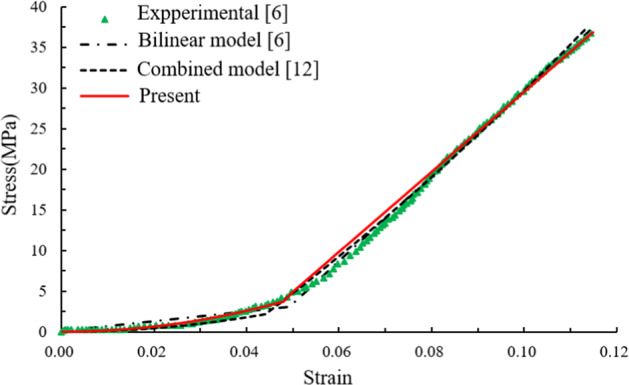
Constitutive behavior of the rabbit medial collateral ligament

Dependence of stress on strain for rabbit medial collateral ligament was obtained by axial tension tests [6], as the green dots shown in Fig. 3.

This curve was even studied by using the bilinear model in [6], and can also be predicted by using the combined model in Eq. (1) and the piecewise constitutive relation in Eq. (3). By using the LMS algorithm, the constitutive parameters are identified for different models and tabulated in Table 2.

**Table 2:** The identified parameters for the rabbit medial collateral ligament

Parameters	*k*_1_ (MPa)	*n*	*ε*_c_	*ε*_th_	*E* (MPa)
Bilinear model	–	–	0.049	–	525.4
Combined model	1149	1.01	0.044	–	330.4
Present	1718	1.02	0.048	0.040	492.1

The modulus is 525.4 MPa for the bilinear model in [6], 330.4 MPa for the combined model, and 492.1 MPa for the piecewise relation. Compared with the group modulus of 469.5 ± 85.23 MPa [6], the piecewise relation gives a more accurate modulus than the others.

In the bilinear model, the critical strain determined by interesting the two linear relations is *ε*_*c*_ = 0.049 from Fig. 3 in [6] after the two linear relations were individually fitted. The value is *ε*_*c*_ = 0.048 for the piecewise relation and *ε*_*c*_ = 0.044 for the combined relation. These three values are almost same.

From Table 2, the value for parameter *n* is almost same as well.

However, the differences are nearly 50% for the parameter *k*_1_ between the piecewise relation and the combined model.

Overall, the piecewise model is better than the others.

## Conclusions

In this paper, a piecewise constitutive model is proposed and compared. The present model is composed of one nonlinear part and another linear part, responsible for the heel region and the linear region of stress–strain curves, respectively. For a given experimental curve, the present model can more precisely predict the two key material parameters (i.e., the moduli and the critical strain) for the collagen fiber-based tissues, as well as other parameters. Based on the philosophy that the constitutive equation of a material can only be determined by experiments [18], the present piecewise constitutive model is justified.

Along this piecewise guideline, a complete constitutive relation can be established for the entire experimental curve of collagen tissues with the distinct failure region by replacing the linear relation for the linear region with a nonlinear relation for both of linear region and failure region. Besides, the present model is a one-dimension relation, and hence the extension to high-order dimensions for general deformation of soft tissues can be further conducted if necessary.

## Footnotes

1 The original form of Eq. (1) in [12] is not correct from the mathematical standpoint.
